# Symptoms predictive of *Fusobacterium necrophorum* pharyngotonsillitis – an observational study of cases presenting to hospitals in Southern Sweden

**DOI:** 10.1007/s10096-024-04827-6

**Published:** 2024-04-12

**Authors:** Josefina Pagels, Gustav Torisson, Lisa Wasserstrom, Katarina Hedin, Karin Holm, David Nygren

**Affiliations:** 1 Division of Infection Medicine, Department of Clinical Sciences, Lund University, Lund, Sweden; 2 Department of Infectious Diseases, Skåne University Hospital, Lund/Malmö, Sweden; 3 Clinical Infection Medicine, Department of Translational Medicine, Lund University, Malmö, Sweden; 4 Department of Clinical Microbiology, Infection Control and Prevention, Skåne University Hospital, Lund, Sweden; 5 Clinical Microbiology, Department of Translational Medicine, Faculty of Medicine, Lund University, Malmö, Sweden; 6 Futurum, Jönköping, Region Jönköping County Sweden; 7 Department of Health, Medicine and Caring Sciences, Linköping University, Linköping, Sweden; 8 Department of Clinical Sciences in Malmö, Family Medicine, Lund University, Malmö, Sweden

**Keywords:** Pharyngotonsillitis, *Fusobacterium necrophorum*, Sore throat, Testing criteria, Clinical score

## Abstract

**Objectives:**

*Fusobacterium necrophorum* is a common cause of pharyngotonsillitis. However, no guidelines exist on when to diagnose or treat it. We aimed to investigate associations between clinical criteria and *F. necrophorum-*positivity in pharyngotonsillitis and assess the predictive potential of a simple scoring system.

**Methods:**

Pharyngotonsillitis patients who were tested for *F. necrophorum* (PCR) and presented to hospitals in the Skåne Region, Sweden, between 2013–2020 were eligible. Data were retrieved from electronic chart reviews and registries. By logistic regression we investigated associations between *F. necrophorum*-positivity and pre-specified criteria: age 13–30 years, symptom duration ≤ 3 days, absence of viral symptoms (e.g. cough, coryza), fever, tonsillar swelling/exudate, lymphadenopathy and CRP ≥ 50 mg/L. In secondary analyses, associated variables were weighted by strength of association into a score and its predictive accuracy of *F. necrophorum* was assessed.

**Results:**

Among 561 cases included, 184 (33%) had *F. necrophorum,* which was associated with the following criteria: age 13–30, symptom duration ≤ 3 days, absence of viral symptoms, tonsillar swelling/exudate and CRP ≥ 50 mg/L. Age 13–30 had the strongest association (OR5.7 95%CI 3.7–8.8). After weighting, these five variables had a sensitivity and specificity of 68% and 71% respectively to predict *F. necrophorum*-positivity at the proposed cut-off.

**Conclusion:**

Our results suggest that *F. necrophorum* cases presenting to hospitals might be better distinguished from other pharyngotonsillitis cases by a simple scoring system presented, with age 13–30 being the strongest predictor for *F. necrophorum*. Prospective studies, involving primary care settings, are needed to evaluate generalisability of findings beyond cases presenting to hospitals.

**Supplementary Information:**

The online version contains supplementary material available at 10.1007/s10096-024-04827-6.

## Introduction

*Fusobacterium necrophorum* is a common pharyngotonsillitis pathogen, found in 21% of pharyngotonsillitis cases overall [1], and in up to 48% of non-streptococcal cases in a study selecting patients aged 18–32 years [2]. Furthermore, a recent study highlighted similar complication rates when *F. necrophorum* or group A streptococci (GAS) were identified [3] and *F. necrophorum* is the most frequent cause of peritonsillar abscesses [4]. While the incidence of invasive *F. necrophorum* disease remains low, including severe manifestations such as Lemierre´s syndrome, it is increasing [5–8].

In a nationwide study in Sweden, a majority of cases presenting with Lemierre’s syndrome or other severe *F. necrophorum* infections had a prior medical visit, which rarely resulted in effective antibiotic therapy [6]. Consequently, it could be argued that current guidelines [9–11] for testing in tonsillitis may need to be revised, as they barely mention *F. necrophorum*. However, as of yet, antibiotic efficacy in *F. necrophorum* pharyngotonsillitis has not been evaluated. In addition, tonsillar carriage rates of *F. necrophorum* have varied between 0–21% [2, 12–18] in studies including adolescents and young adults, complicating the interpretation of a positive finding. Nevertheless, the tonsillar carriage rate of GAS (by PCR) in similar age groups have been found to be 9% [12].

When pharyngotonsillitis is suspected, current Swedish practice [9] recommend using the presence of three or more Centor criteria [19] to guide testing with a Rapid antigen detection test (RADT) for GAS. These criteria include a history of fever, tonsillar exudate, cervical lymphadenopathy, and absence of cough [9, 19]. Another common score, the FeverPAIN-score [20], includes fever in the last 24 h, tonsillar exudate, symptom duration ≤ 3 days, severely inflamed tonsils, and absence of cough and coryza, with suggested antibiotic treatment for patients fulfilling four or more criteria [21]. Unsurprisingly, since they were developed to detect beta-haemolytic streptococci [19–21], they have been found to have inferior accuracy to detect *F. necrophorum* [13, 14, 16]. No score has been developed with the purpose to detect *F. necrophorum*.

The primary aim of this study was to investigate the association of clinically easily available variables with *F. necrophorum*-positivity among patients with pharyngotonsillitis presenting to hospital. The secondary aim was to investigate the predictive ability of these pre-specified criteria of *F. necrophorum*-positivity. Their accuracy to predict *F. necrophorum* were expected to outperform previous criteria developed for beta-haemolytic streptococci in pharyngotonsillitis, e.g., Centor [19] and FeverPAIN-scores [20, 21]. This expectation was partly based on the assumption that among patients referred to hospitals, severe presentations yet with negative RADT, or cases with severe, yet atypical presentations, would be overrepresented, making established criteria less specific.

## Method

### Study design and setting

In this retrospective study, all pharyngotonsillitis cases tested for *F. necrophorum* with available medical records in the Skåne Region, Sweden were investigated, limiting cases to those presenting to hospitals due to data availability. All nine hospitals in the region were included in the study, and the population was 1.4 million (2021). The study period ranged from June 2013 until December 2020, encompassing the full period during which *F. necrophorum*-PCR and registry data were available in this region. Electronic chart reviews were performed in addition to data collection from registries on antibiotic prescriptions, diagnoses and microbiological findings. The study was approved by the local Ethical Review Board in Lund, Sweden (number 2017/971).

### Participants

Patients diagnosed with acute pharyngotonsillitis (ICD-10-code J02-03) in any hospital (emergency or outpatient department) and tested with PCR for *F. necrophorum* were eligible for the study*.* Patients were excluded if they had a previous (0–30 days) diagnosis of peritonsillar or other pharyngeal abscesses, sinusitis, otitis, chronic pharyngotonsillitis, sepsis or septic complication (ICD-codes in Appendix 1). They were also excluded if they had received antibiotics active against *F. necrophorum* 1–30 days prior to the visit, defined as beta-lactam antibiotics, metronidazole, or clindamycin, or if misclassification of diagnosis was discovered upon chart review. The follow up period for each patient was 30 days from the index visit. The same person could be included again if at least 30 days had followed since the previous inclusion. All PCR-positive cases for *F. necrophorum* were defined as *F. necrophorum* regardless of the presence of a co-infection.

### Variables and statistical analysis

The following criteria were pre-specified and associations with *F. necrophorum* were investigated:

- Age 13–30 years (0/1)
- Symptom duration ≤ 3 days (0/1)
- Absence of common cold symptoms (e.g. cough, coryza, conjunctivitis) (0/1)
- Fever ≥ 38° C (including anamnestic fever) (0/1)
- Tonsillar swelling or exudate (0/1)
- Cervical lymphadenopathy (0/1)
- CRP ≥ 50 mg/L (0/1)

Variables were chosen a priori, based on literature reviews of known clinical characteristics of patients with bacterial pharyngotonsillitis, including *F. necrophorum*, and expert opinion [5–7, 14, 19, 20, 22]. Information about patient characteristics, symptoms, comorbidities, and microbiological findings were collected from the chart. When defining variables of the Centor and FeverPAIN-scores, absence of cough was equalled to absence of viral symptoms, fever was defined as either measured temperature ≥ 38° or anamnestic fever, and severely inflamed tonsils were defined as tonsillar swelling or exudate. Continuous variables were described as median with interquartile range provided. Dichotomous variables were described as counts and percentages.

As part of the study design, a pilot study including 25 patients was performed to evaluate data quality and data missingness after pre-specifying criteria. Handling of missing data was pre-defined. Clinical signs and symptoms were considered as absent if not described in the chart review. If < 5% were missing for any of the pre-specified criteria, complete case analysis was performed. If ≥ 5% of data were missing (only CRP), multiple imputation (25 iterations) with predictive mean matching (5 nearest neighbours) was performed with a continuous output. Pre-specified variables and *F. necrophorum-*PCR were used as independent variables. Data were considered missing at random, with similar rates missing in patients regardless of *F. necrophorum* PCR-positivity. Sensitivity analyses using complete case analysis as comparison were performed. Following multiple imputation of CRP as a continuous variable, estimates from the 25 iterations were dichotomized (≥ 50 mg/L (0/1)) and then pooled.

For the primary analysis, logistic regression analyses were performed to evaluate associations between *F. necrophorum*-positivity and pre-specified variables, with the reference category being cases with a negative *F. necrophorum*-PCR. Crude and adjusted odds ratios (OR) were presented. A sensitivity analysis was performed to compare cases with monomicrobial *F. necrophorum* infection with those with co-infection.

All eligible cases during the period with available data were included, determining the sample size. With seven dichotomous variables (7 degrees of freedom), we expected a sample size with > 15 events per variable included, lowering the risk of overfitting. Statistical analyses were performed using Stata: Release 17.

### Secondary analysis

In the secondary analysis, the variables associated with *F. necrophorum* in the crude analysis were weighted according to strength of association, creating a weighted predictive model, with a simple scoring system. At each point score of this model, its sensitivity, specificity and positive predictive value were tabulated. The predictive model was then assessed against existing scores previously developed to identify beta-haemolytic streptococci, i.e., the Centor [19] and FeverPAIN-scores [20, 21]. The ability of these three predictive models to detect *F. necrophorum* or any bacterial infection was visualized by ROC-curves and tabulated with suggested cut-offs.

## Results

**Fig. 1:**
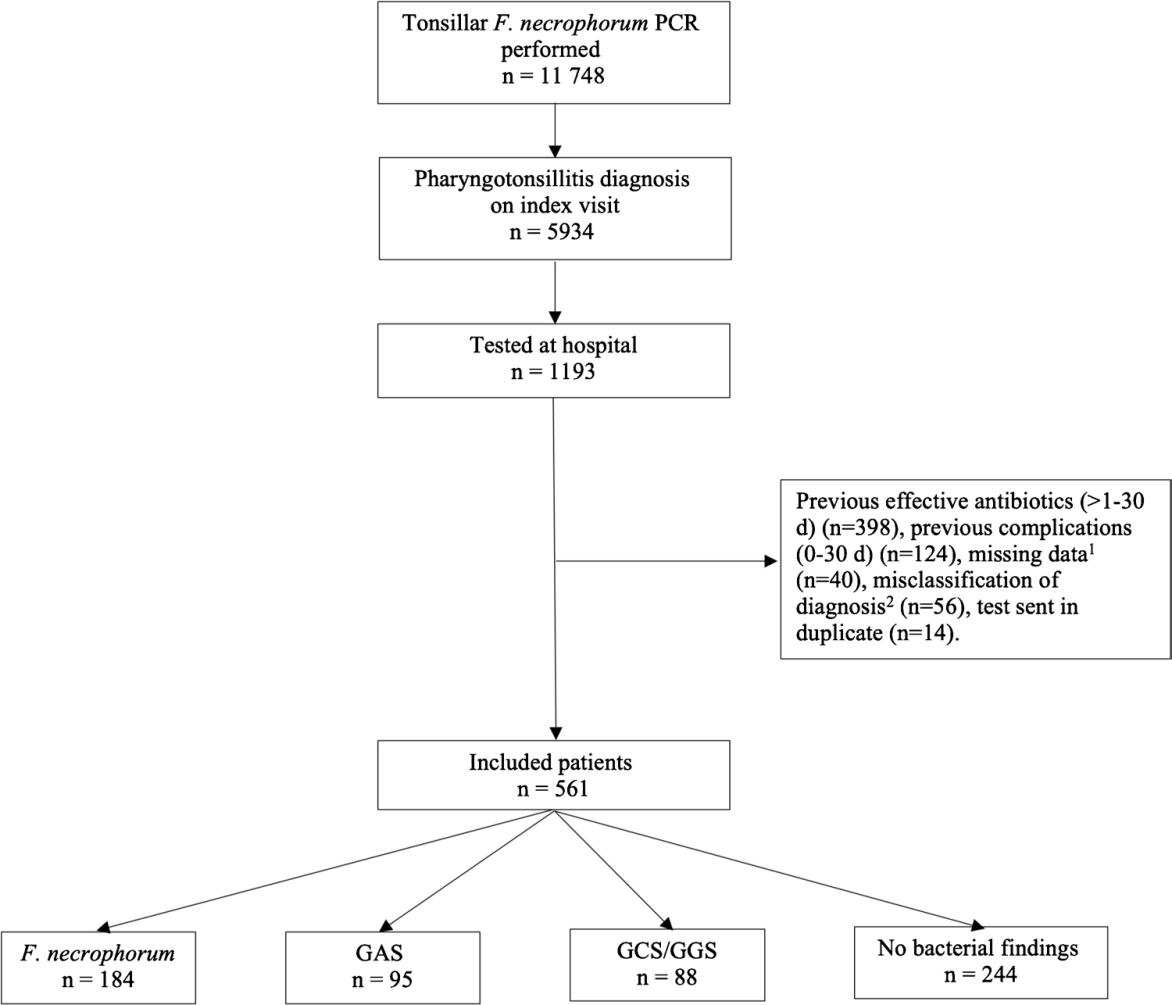
Flowchart showing the selection process of inclusion of eligible patients by case definition and exclusion criteria for this study, with the microbial findings (F. necrophorum, Group A streptococci (GAS), Group C/G streptococci (GCS/GGS) or no bacterial findings) presented. If more than one finding was made, the case is represented in more than one box. ^1^ 9 cases had missing medical charts, 27 cases had no documented symptom duration, 3 cases had no documented throat status and in 1 case the F. necrophorum sample had not been analysed. ^2^15 cases lacked tonsils (previous tonsillectomy), 4 cases had lingual tonsillitis, 37 cases had a symptom duration of > 14 days

Of a total of 11,748 tests for *F. necrophorum* performed from throat swabs in the Skåne Region during the study period, 1193 were eligible for the study, since they were performed in pharyngotonsillitis cases who presented to hospitals. 561 were included, with 632 fulfilling exclusion criteria (Fig. 1). 184 (33%) tested positive for *F. necrophorum,* 95 (17%) tested positive for GAS either by culture or RADT and 88 (16%) tested positive for group C or G streptococci (GCS or GGS). 244 (43%) had no bacterial findings, in 9 of these cases (2% of the total study population), no culture for beta-haemolytic streptococci was performed (Table 1). Of the eligible cases, less than 5% had missing data on symptom duration or throat status and were excluded. CRP levels were missing in 21% and were imputed, dichotomized (≥ 50 mg/L (0/1)), and then pooled.

**Table 1:** Baseline characteristics of patients presenting with pharyngotonsillitis to hospitals with F. necrophorum-PCR performed, grouped by F. necrophorum, Group A streptococci (GAS), Group C/G streptococci (GCS/GGS) or negative bacterial findings. If more than one bacterial finding was found, each case is represented in more than one column. In nine cases no culture had been performed, these were considered as negative unless positive for F. necrophorum (3 cases) or RADT (0 cases). Numbers are presented as N (%). N/total (%) is presented when any data were missing

	*F. necrophorum* *n* = 184	GAS *n* = 95	GCS/GGS *n* = 88	Negative *n* = 244
Age (median, IQR)	21 (17–28)	29 (17–40)	19 (17–25)	19 (10–37)
Age 13–30 years, %	153 (83%)	38 (40%)	71 (81%)	106 (43%)
Female, %	114 (62%)	52 (55%)	60 (68%)	122 (50%)
Any comorbidity^1^, %	26 (14%)	18 (19%)	13 (15%)	59 (24%)
CRP (complete case) (median, IQR), mg/L	124 (57–190)	114 (50–193)	123 (66–200)	73 (24–119)
CRP (post-imputation)^2^ (median, IQR), mg/L	119 (56–186)	107 (44–185)	110 (55–187)	71 (24–120)
CRP ≥ 50 mg/L (complete case)	119/149 (80%)	60/80 (75%)	57/70 (81%)	110/190 (58%)
CRP ≥ 50 mg/L (post-imputation)^2^	145 (79%)	68 (72%)	67 (77%)	141 (58%)
Centor criteria (median, IQR)	3 (3–4)	3 (2–4)	3 (3–4)	3 (2–4)
FeverPAIN-score (median, IQR)	4 (3–5)	4 (3–4)	4 (3–5)	3 (3–4)
Microbial findings	*F. necrophorum* *n* = 184	GAS *n* = 95	GCS/GGS *n* = 88	Negative *n* = 244
*F. necrophorum*, %	184 (100%)	9 (10%)	41 (47%)	0%
RADT-positive, %	5/135 (4%)	52/72 (72%)	0/72 (0%)	0/180 (0%)
GAS (culture), %	9/181 (5%)	87 (92%)	0%	0/238 (0%)
GAS (RADT or culture), %	9 (5%)	95 (100%)	0%	0/242 (0%)
GCS/GGS, %	41/181 (23%)	0%	88 (100%)	0/238 (0%)
Signs and symptoms	*F. necrophorum* *n* = 184	GAS *n* = 95	GCS/GGS *n* = 88	Negative *n* = 244
Symptom duration ≤ 3 days	126 (69%)	60 (63%)	55 (63%)	127 (52%)
Absence of viral symptoms^3^	164 (89%)	78 (82%)	72 (82%)	189 (78%)
Fever ≥ 38° C or anamnestic fever	139 (76%)	75 (79%)	71 (81%)	163 (67%)
Tonsilar swelling or exudate	172 (94%)	72 (76%)	80 (91%)	201 (82%)
Lymphadenopathy	101 (55%)	57 (60%)	52 (59%)	131 (54%)

^1^ According to the Charlson comorbidity index [23]

^2^ 21% had missing data. When missing, data was imputed using multiple imputation (25 iterations) with predictive mean matching

^3^ Viral symptoms defined as cough, coryza, or conjunctivitis

### Baseline characteristics

The median age of the patients with *F. necrophorum* was 21 years. A vast majority was 13–30 years. The age distribution in the GCS/GGS group was similar, while less than half of the patients with GAS were between 13–30 years. A slight majority of the *F. necrophorum-*positive patients were female and regardless of microbial findings, comorbidities were rare (Table 1).

### Primary analysis results

Of the seven pre-specified variables investigated, age 13–30 years (OR 5.7 (95% confidence interval (CI) 3.7–8.8)), symptom duration ≤ 3 days (OR 1.8 (95% CI 1.3–2.7)), absence of viral symptoms (OR 2.2 (95% CI 1.3–3.8)), tonsillar swelling or exudate (OR 3.3 (95% CI 1.8–6.3)) and CRP ≥ 50 mg/L (OR 2.2 (95% CI 1.3–3.6)) were associated with *F. necrophorum*-positivity in the crude analysis, with age having the strongest association. Lymphadenopathy (OR 1.0 (95% CI 0.7–1.4)) and fever (OR1.3 (95% CI 0.8–1.9)) were not associated with *F. necrophorum*-positivity. In the multivariate analysis, all associations remained similar (Table 2), and sensitivity analyses had similar results (Appendix 2, Tables 1, 2, 3, 4).

**Table 2:** Crude and adjusted odds ratio for associations between F. necrophorum PCR-positivity in pharyngotonsillitis and pre-specified variables (0/1), calculated by logistic regression with Odds Ratio (OR) and 95% confidence intervals (95%CI) provided. The reference category was patients with negative F. necrophorum-PCR. Weighted points in the predictive model depending on strength of associations are presented

	Crude OR (95%CI) for *F. necrophorum*	Adjusted OR (95%CI) for *F. necrophorum*	Weighted points in predictive model
Age 13–30 years	5.7 (3.7–8.8)	5.6 (3.6–8.8)	3 p
Tonsillar swelling or exudate	3.3 (1.8–6.3)	2.4 (1.2–4.8)	1 p
CRP ≥ 50 mg/L^1^	2.2 (1.3–3.6)	2.2(1.2–3.8)	1 p
Absence of viral symptoms^2^	2.2 (1.3–3.8)	2.3 (1.3–4.1)	1 p
Symptom duration ≤ 3 days	1.8 (1.3–2.7)	1.6 (1.1–2.5)	1 p
Fever ≥ 38° C or anamnestic fever	1.3 (0.8–1.9)	1.1 (0.6–1.6)	Not included
Lymphadenopathy	1.0 (0.7–1.4)	0.9 (0.6–1.3)	Not included
Maximum points of predictive model			7 p

^1^ 21% had missing data. When missing, data was imputed using multiple imputation (25 iterations) with predictive mean matching

^2^ Viral symptoms were defined as cough, coryza, or conjunctivitis

### Secondary analyses

**Fig. 2:**
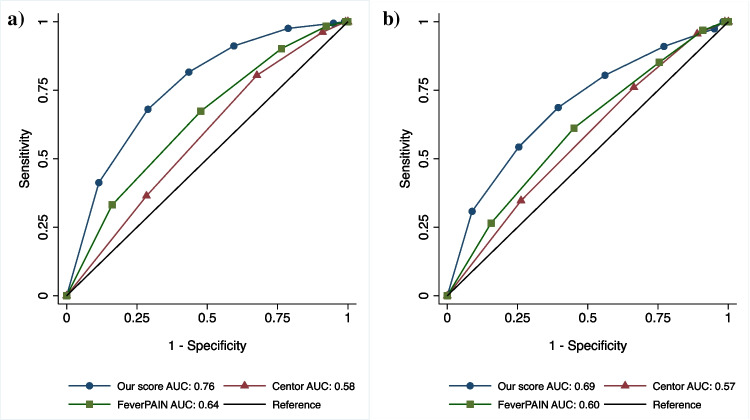
**a**-**b**: Receiver operating characteristic (ROC) curve highlighting the performance of our model in comparison with the Centor criteria and the FeverPAIN-score in predicting F. necrophorum-positivity (2a) or any microbial findings (2b) among pharyngotonsillitis patients

For the scoring system, variables associated with *F. necrophorum* were weighted by strength of association. Age 13–30 years was assigned 3 points, while symptom duration ≤ 3 days, absence of viral symptoms, tonsillar swelling or exudate and CRP ≥ 50 mg/L were assigned one point each with a maximum of seven points (Table 2). Sensitivity and specificity to detect *F. necrophorum* were calculated at each point-score. At a cut-off level of six points in this cohort, the *F. necrophorum*-prevalence (positive predictive value) was 53% above and 18% below. As expected, lower positive predictive values were seen for Centor criteria and FeverPAIN-score (Table 3), which performed worse at identifying both *F. necrophorum* or any bacterial infection (Fig. 2).

**Table 3:** Sensitivity and specificity at different cut-offs for prediction of F. necrophorum-positivity in pharyngotonsillitis patients using the predictive model presented in this paper, the FeverPAIN-score or the Centor criteria. The suggested cut-off level for our model, as well as the previously proposed and established cut-offs for the FeverPAIN-score and the Centor criteria are highlighted and indicated by the marked lines. The total numbers of patients testing positive for F. necrophorum above or below these cut-offs are presented

Our model	Sensitivity	Specificity	F. necrophorum-positivity above and below cut-off level
*1* ≥ *points*	100%	1%	59/327 (18%)
*2* ≥ *points*	99%	5%	
*3* ≥ *points*	98%	21%	
*4* ≥ *points*	91%	41%	
*5* ≥ *points*	82%	57%	
***6* ≥ *points***	68%	71%	125/234 (53%)
*7 points*	41%	89%	
FeverPAIN Score			
*1* ≥ *criteria*	100%	0.8%	60/257 (23%)
*2* ≥ *criteria*	98%	8%	
*3* ≥ *criteria*	90%	24%	
***4* ≥ *criteria***	67%	52%	124/304 (41%)
*5 criteria*	33%	83%	
Centor criteria			
*1* ≥ *criteria*	100%	0.8%	36/158 (23%)
*2* ≥ *criteria*	96%	9%	
***3* ≥ *criteria***	80%	32%	148/403 (37%)
*4 criteria*	36%	72%

## Discussion

We investigated signs and symptoms in hospital cases with *F. necrophorum-*pharyngotonsillitis to describe how their presentation may distinguish them from patients with other causes of pharyngotonsillitis. Of seven pre-specified variables*,* five were associated with *F. necrophorum*-PCR-positivity, i.e., age 13–30 years, symptom duration of ≤ 3 days, absence of viral symptoms, tonsillar swelling or exudate and CRP ≥ 50 mg/L*.* After weighting criteria by strengths of associations, a predictive model was evaluated and at a cut-off of six or more of seven points, every second patient with pharyngotonsillitis had a positive *F. necrophorum*-PCR.

The variables investigated were inspired by previous studies [1, 14] and scores used in pharyngotonsillitis, yet varied slightly from the Centor [19] and FeverPAIN-scores [20, 21] suggesting that *F. necrophorum* could be distinguished from other causes of pharyngotonsillitis*.* Most importantly, the age criterion was the strongest predictor of *F. necrophorum*, suggesting that focusing on adolescents and young adults is advisable in future guidelines on *F. necrophorum*-pharyngotonsillitis, specifically in RADT-negative cases presenting to hospital. No randomized controlled trial has investigated antibiotic therapy in *F. necrophorum-*pharyngotonsillitis. Yet, a Cochrane meta-analysis on antibiotic treatment in pharyngotonsillitis patients with negative cultures for beta-haemolytic streptococci have shown symptom reduction following exposure to antibiotics [24]. It is conceivable that this could also apply to *F. necrophorum*, although studies are needed.

No previous criteria have been developed to predict *F. necrophorum.* However, the performance of the Centor criteria in predicting *F. necrophorum* pharyngotonsillitis has previously been summarized in a meta-analysis by Klug et al [1], focused on three prospective studies with low risk of bias performed in a primary care setting [13, 14, 16]. Based on data from this meta-analysis, a cut-off of three or more Centor criteria would only identify 30% of patients with *F. necrophorum*. Conversely, in the large data set presented in our study, 80% of patients with *F. necrophorum* had three or more Centor criteria. Presumably, among the patients referred to hospital in this study, many likely had high Centor criteria, negative RADT, yet severe or atypical presentations. Consequently, regardless of microbiological findings, most patients enrolled in our study had Centor criteria of three or more (72%). Hence, in our cohort of hospital cases the Centor criteria had a low positive predictive value due to low specificity (Table 3). First, this highlight that our data represent a severely ill pharyngotonsillitis cohort, most with high Centor scores. Second, given the retrospective nature of data, selection bias is likely to occur since current practice in Sweden [9] (focused on Centor criteria) will likely select cases with high Centor criteria or severely ill cases for further testing despite a negative RADT. The previously cited meta-analysis [1] highlights that Centor criteria perform worse in detecting *F. necrophorum* efficiently when compared to GAS in the primary health care setting. Our study shows that the Centor and FeverPAIN scores have worse performance to predict *F. necrophorum* among hospital cases compared to our proposed criteria, yet we can draw no firm conclusions on their performance in the primary care setting. As neither were developed to predict *F. necrophorum*, this is expected, yet indicates the need for a different tool to decide whom to test for *F. necrophorum*. The findings of this paper should encourage the validation of our score, which shows promise among cases presenting to hospital. However, its sensitivity and specificity of 68 and 71% highlights room for improvement. Meanwhile, meta-analyses of validation studies on criteria used to identify GAS in pharyngotonsillitis have similarly shown moderate accuracy in both primary care [25] and hospitals [26]. Nevertheless, these criteria remain useful. Importantly, due to the known asymptomatic tonsillar carriage of *F. necrophorum* and GAS [12, 18], the aim of clinical criteria in pharyngotonsillitis should not be to identify all cases positive for *F. necrophorum* or GAS, but preferably target cases most likely to benefit from treatment.

There are several limitations to this study. As stated, it suffers from selection bias since it only includes patients presenting to hospital. In Swedish practice pharyngotonsillitis patients with three or more Centor criteria are recommended to be tested with a RADT for GAS [9]. Extensive diagnostics for other beta-haemolytic streptococci or *F. necrophorum* is mentioned in severely ill patients or patients with persistent or worsening symptoms after three days. Hence, the patients in this study are both selected since they present to hospital and since the case definition requires testing. In addition, most patients testing positive for GAS using RADT would likely not have been tested any further, decreasing the number of GAS-patients included. Thus, generalizability of the findings is limited to severe and mainly RADT-negative pharyngotonsillitis cases presenting to hospital. Nonetheless, these are the patients where current practice recommendations [9] mention extended diagnostics beyond an RADT. Important to note, the reference category in regression analyses is defined by a negative *F. necrophorum*-PCR, and not negative results for any bacteria, since the aim was to distinguish *F. necrophorum-*pharyngotonsillitis from all other causes, including beta-haemolytic streptococci. By highlighting distinctive features of *F. necrophorum* pharyngotonsillitis in a large number of severely ill cases with pharyngotonsillitis, future guidelines are aided to provide better guidance, with the main predictor being age 13–30 years. Guidelines on management of pharyngotonsillitis might need revision following evidence during the last ten years establishing *F. necrophorum* as an important cause of pharyngotonsillitis [1, 3], peritonsillar abscess [4, 27] and with increasing incidence of invasive infections in adolescents and young adults [6].

Future prospective studies are needed to investigate associations of these and other clinically easily available variables to predict *F. necrophorum*-positivity. Preferably, studies should be performed in a primary care population, where most cases of pharyngotonsillitis present. It is likely that the sensitivity of the predictive model here discussed will decrease considerably if used in a less severely ill population and would likely require lower cut-offs or potentially investigate other variables. Nevertheless, in the sub-group of patients with severe pharyngotonsillitis and negative RADT for GAS, signs and symptoms presented here could help guide physicians on whom to test for *F. necrophorum*. Investigating whether treatment leads to reduced symptoms and complications remains necessary, as do the development of more easily available tests for *F. necrophorum*, preferably an RADT.

Given the increased acknowledgement and importance of *F. necrophorum* in pharyngotonsillitis in high-income countries, guidance on whom and when to test for it is needed. In a large but selected cohort of severe cases of pharyngotonsillitis presenting to hospital, age between 13–30 was identified as the strongest predictor of *F. necrophorum*-positivity, followed by tonsillar swelling or exudate, CRP ≥ 50 mg/L, absence of viral symptoms and symptom duration ≤ 3 days. When used to predict presence of *F. necrophorum*, these easily available clinical characteristics outperformed previous established criteria, but need to be investigated and likely finetuned in primary health care populations prior to clinical use.

### Supplementary Information

Below is the link to the electronic supplementary material.

Supplementary file1 (DOCX 37 KB)

## Data Availability

According to the ethical approval by the Ethical Review Board in Lund, Sweden (number 2017/971) data is not allowed to be shared publicly. For specific questions regarding the data, please contact the authors.
