# Supplementary material for: Symptoms predictive of Fusobacterium necrophorum pharyngotonsillitis – an observational study of cases presenting to hospitals in Southern Sweden
Source: Eur J Clin Microbiol Infect Dis. 2024 Apr 12;43(6):1099–107. doi: 10.1007/s10096-024-04827-6 (PMC11178599; doi:10.1007/s10096-024-04827-6)
Supplement: Supplementary file 1 — Supplementary file1 (DOCX 37 KB) [file 10096_2024_4827_MOESM1_ESM.docx]

# **Appendix 1**

**List of ICD-codes defining inclusion and exclusion criteria and complications**

|  | | | |
| --- | --- | --- | --- |
| **Eligibility codes for inclusion on first visit:** | | | |
|  |  |  |  |
| **Diagnosis code (ICD-10)** | **Description** | **Diagnosis code, primary healthcare** | **Description, primary healthcare (KSH-97)** |
| J02- | Acute pharyngitis | J02- | Acute pharyngitis |
| J020 | Streptococcal pharyngitis | J02- | Acute pharyngitis |
| J028 | Acute pharyngitis due to other specified organisms | J02- | Acute pharyngitis |
| J029 | Acute pharyngitis, unspecified | J02- | Acute pharyngitis |
| J03- | Acute tonsillitis | J03- | Acute tonsillitis |
| J030 | Streptococcal tonsillitis | J03- | Acute tonsillitis |
| J038 | Acute tonsillitis due to other specified organisms | J03- | Acute tonsillitis |
| J039 | Acute tonsillitis, unspecified | J03- | Acute tonsillitis |
|  |  |  |  |

**Codes defining exclusion criteria (prior 30 days including day of index visit):**

| **Diagnosis code (ICD-10)** | **Description** | **Diagnosis code, primary healthcare** | **Description, primary healthcare (KSH-97)** |
| --- | --- | --- | --- |
|  |  |  |  |
| **Peritonsillar abscess** | |  |  |
| J36- | Peritonsillar abscess | J36- | Peritonsillar abscess |
| J369 | Peritonsillar abscess | J36- | Peritonsillar abscess |
|  |  |  |  |
| **Other pharyngeal abscess** | |  |  |
| J39.0 | Retropharyngeal and parapharyngeal abscesss | J39.0 | Retropharyngeal abscess |
| J39.1 | Other pharyngeal abscess | J39.1 | Pharyngeal absess |
|  |  |  |  |
| **Chronic tonsillitis** | |  |  |
| J350 | Chronic tonsillitis | J312P | Chronic pharyngitis |
| J312 | Chronic pharyngitis | J312P | Chronic pharyngitis |
| J359 | Chronic disease in tonsils and adenoids | J359P | Chronic disease in tonsils and adenoids |
|  |  |  |  |
| **Sinusitis** |  |  |  |
| J01- | Acute sinusitis | J01- | Acute sinusitis |
| J010 | Acute maxillary sinusitis | J01- | Acute sinusitis |
| J011 | Acute frontal sinusitis | J01- | Acute sinusitis |
| J012 | Acute ethmoidal sinusitis | J01- | Acute sinusitis |
| J013 | Acute sphenoidal sinusitis | J01- | Acute sinusitis |
| J014 | Acute pansinusitis | J01- | Acute sinusitis |
| J018 | Other acute sinusitis | J01- | Acute sinusitis |
| J019 | Acute sinusitis, unspecified | J01- | Acute sinusitis |
|  |  |  |  |
| **Otitis** | |  |  |
| H660 | Acute suppurative otitis media | H660 | Acute suppurative otitis media |
| H664 | Suppurative otitis media, unspecified | H669P | Otitis media, unspecified |
| H669 | Otitis media, unspecified | H669P | Otitis media, unspecified |
| H669P | Otitis media, unspecified | H669P | Otitis media, unspecified |
|  |  |  |  |

**Codes defining complications (within 30 days):**

| **Diagnosis code (ICD-10)** | **Description** | **Diagnosis code, primary healthcare** | **Description, primary healthcare (KSH-97)** |
| --- | --- | --- | --- |
| **Peritonsillar abscess** | |  |  |
| J36- | Peritonsillar abscess | J36- | Peritonsillar abscess |
| J369 | Peritonsillar abscess | J36- | Peritonsillar abscess |
|  |  |  |  |
| **Other pharyngeal abscess** | |  |  |
| J39.0 | Retropharyngeal and parapharyngeal abscesss | J39.0 | Retropharyngeal abscess |
| J39.1 | Other pharyngeal abscess | J39.1 | Pharyngeal absess |
|  |  |  |  |
| **Recurrence of pharyngotonsillitis d 15-30** | | |  |
| J02- | Acute pharyngitis | J02- | Acute pharyngitis |
| J020 | Streptococcal pharyngitis | J02- | Acute pharyngitis |
| J028 | Acute pharyngitis due to other specified organisms | J02- | Acute pharyngitis |
| J029 | Acute pharyngitis, unspecified | J02- | Acute pharyngitis |
| J03- | Acute tonsillitis | J03- | Acute tonsillitis |
| J030 | Streptococcal tonsillitis | J03- | Acute tonsillitis |
| J038 | Acute tonsillitis due to other specified organisms | J03- | Acute tonsillitis |
| J039 | Acute tonsillitis, unspecified | J03- | Acute tonsillitis |
|  |  |  |  |
| **Sinusitis** |  |  |  |
| J01- | Acute sinusitis | J01- | Acute sinusitis |
| J010 | Acute maxillary sinusitis | J01- | Acute sinusitis |
| J011 | Acute frontal sinusitis | J01- | Acute sinusitis |
| J012 | Acute ethmoidal sinusitis | J01- | Acute sinusitis |
| J013 | Acute sphenoidal sinusitis | J01- | Acute sinusitis |
| J014 | Acute pansinusitis | J01- | Acute sinusitis |
| J018 | Other acute sinusitis | J01- | Acute sinusitis |
| J019 | Acute sinusitis, unspecified | J01- | Acute sinusitis |
|  |  |  |  |
| **Otitis** | |  |  |
| H660 | Acute suppurative otitis media | H660 | Acute suppurative otitis media |
| H664 | Suppurative otitis media, unspecified | H669P | Otitis media, unspecified |
| H669 | Otitis media, unspecified | H669P | Otitis media, unspecified |
| H669P | Otitis media, unspecified | H669P | Otitis media, unspecified |
|  |  |  |  |
|  |  |  |  |
| **Sepsis or septic complications** | |  |  |
| A41-P | Other sepsis | A41-P | Septicaemia |
| A419 | Sepsis, unspecified | A41-P | Septicaemia |
| A418 | Other specified sepsis | A41-P | Septicaemia |
| A400 | Sepsis due to streptococcus, group A | A41-P | Septicaemia |
| A414 | Sepsis due to anaerobic bacteria | A41-P | Septicaemia |
| A415 | Sepsis due to Gram-negative bacteria | A41-P | Septicaemia |
| A408 | Other streptococcal sepsis | A41-P | Septicaemia |
| A409 | Streptococcal sepsis, unspecified | A41-P | Septicaemia |
| A48.3 | Toxic shock syndrome | A41-P | Septicaemia |
| J85- | Lung abscess | A41-P | Septicaemia |
| I26- | Pulmonary embolization (Lemierre’s syndrome) | I-26- | Pulmonary embolism |
| M726 | Necrotizing fasciitis | A41-P | Septicaemia |

**Appendix 2**

**Supplementary table 1**

*Sensitivity analysis comparing results of the primary analysis, using solely complete case analysis (n=445), excluding all cases with CRP missing (n=116). Associations between F. necrophorum-positivity and pre-specified variables (0/1) are investigated, with odds ratio (OR) and 95% confidence intervals (95%CI) provided for crude and adjusted analyses. The reference category was patients with negative F. necrophorum-PCR*.

| **Supplementary table 1**  **N=445** | **Crude OR (95%CI) for**  ***F. necrophorum*** | **Adjusted OR (95%CI) for *F. necrophorum*** |
| --- | --- | --- |
| **Age 13-30 years** | 6.1 (3.7-9.9) | 6.2 (3.7-10.2) |
| **Tonsillar swelling or exudate** | 2.9 (1.5-5.8) | 2.2 (1.0-4.7) |
| **CRP ≥ 50 mg/L** | 2.2 (1.4-3.5) | 2.2 (1.3-3.8) |
| **Absence of viral symptoms^1^** | 2.1 (1.2-3.7) | 2.2 (1.2-4.2) |
| **Symptom duration ≤ 3 days** | 1.8 (1.2-2.7) | 1.6 (1.0-2.6) |
| **Fever ≥ 38° C or anamnestic fever** | 1.2 (0.8-2.0) | 0.8 (0.5-1.5) |
| **Lymphadenopathy** | 1.0 (0.7-1.5) | 0.9 (0.6-1.4) |
|  |  |  |

*^1^ Viral symptoms were defined as cough, coryza, or conjunctivitis.*

**Supplementary table 2:1-2**

*Sensitivity analysis comparing results of the primary analysis without dichotomization of CRP with (2:1) and without (2:2) imputation of missing CRP. Associations between F. necrophorum PCR-positivity and pre-specified variables (0/1) are investigated, with odds ratio (OR) and 95% confidence intervals (95%CI) provided for crude and adjusted analyses. The reference category was patients with negative F. necrophorum-PCR*.

| **Supplementary table 2:1**  **n=561** | **Crude OR (95%CI) for**  ***F. necrophorum*** | **Adjusted OR (95%CI) for *F. necrophorum*** |
| --- | --- | --- |
| **Age 13-30 years** | 5.7 (3.7-8.8) | 5.7 (3.6-8.9) |
| **Tonsillar swelling or exudate** | 3.3 (1.8-6.3) | 2.3 (1.2-4.7) |
| **CRP mg/L** | 1.004 (1.002-1.007) | 1.004 (1.001-1.007) |
| **Absence of viral symptoms^1^** | 2.2 (1.3-3.8) | 2.4 (1.3-4.2) |
| **Symptom duration ≤ 3 days** | 1.8 (1.3-2.7) | 1.6 (1.1-2.4) |
| **Fever ≥ 38° C or anamnestic fever** | 1.3 (0.8-1.9) | 1.0 (0.6-1.6) |
| **Lymphadenopathy** | 1.0 (0.7-1.4) | 0.9 (0.6-1.3) |
|  |  |  |
|  |  |  |

| **Supplementary table 2:2**  **n=445** | **Crude OR (95%CI) for**  ***F. necrophorum*** | **Adjusted OR (95%CI) for *F. necrophorum*** |
| --- | --- | --- |
| **Age 13-30 years** | 6.1 (3.7-9.9) | 6.3 (3.8-10.4) |
| **Tonsillar swelling or exudate** | 2.9 (1.5-5.8) | 2.1 (1.0-4.5) |
| **CRP mg/L** | 1.004 (1.002-1.007) | 1.004 (1.002-1.007) |
| **Absence of viral symptoms^1^** | 2.1 (1.2-3.7) | 2.3 (1.2-4.3) |
| **Symptom duration ≤ 3 days** | 1.8 (1.2-2.7) | 1.6 (1.0-2.5) |
| **Fever ≥ 38° C or anamnestic fever** | 1.2 (0.8-2.0) | 0.9 (0.5-1.6) |
| **Lymphadenopathy** | 1.0 (0.7-1.5) | 0.8 (0.5-1.3) |
|  |  |  |
|  |  |  |

**Supplementary table 3**

*F. necrophorum-positivity in patients with or without missing data (CRP).*

| **Supplementary Table 3** | **No missing data**  **(CRP)**  **N=445** | **Missing data (CRP)**  **N=116** |
| --- | --- | --- |
| ***F. necrophorum* PCR-positive, n (%)** | 149 (33%) | 35 (30%) |

**Supplementary table 4**

*Sensitivity analysis investigating differences between patients with F. necrophorum PCR-positivity with and without co-infections with beta-haemolytic strepotococci (GAS, GCS or GGS)*

| **Supplementary Table 4** | **Monomicrobial**  ***F. necrophorum* infection**  **n=134** | **Co-infection with**  **GAS, GCS or GGS**  **n=50** |
| --- | --- | --- |
| **GAS (RADT or culture), %** | 0% | 9 (18%) |
| **GCS/GGS (culture), %** | 0% | 41 (82%) |
|  |  |  |
| **Age (median, IQR)** | 22 (17-28) | 20 (17-29) |
| **Age 13-30 years, %** | 113 (84%) | 40 (80%) |
| **Female, %** | 82 (61%) | 32 (64%) |
| **Any comorbidity^1^, %** | 18 (13%) | 8 (16%) |
| **CRP (complete case) (median, IQR), mg/L** | 117 (56-186) | 131 (75-200) |
| **CRP with imputation (median, IQR), mg/L** | 116 (55-186) | 125 (65-200) |
| **CRP > 50 mg/L (complete case)** | 82/105 (78%) | 37/44 (84%) |
| **CRP > 50 mg/L (imputed)** | 104 (78%) | 41 (81%) |
|  |  |  |
| **Signs and symptoms** | **Monomicrobial**  ***F. necrophorum* infection**  **n=134** | **Co-infection with**  **GAS, GCS or GGS**  **n=50** |
| **Symptom duration ≤ 3 days** | 88 (66%) | 38 (76%) |
| **Absence of viral symptoms^3^** | 121 (90%) | 43 (86%) |
| **Tonsil swelling or exudate** | 125 (93%) | 47 (94)% |
| **Lymphadenopathy** | 70 (52%) | 31 (62%) |
| **Fever ≥ 38° C or anamnestic fever** | 98 (73%) | 41 (82%) |
